# Physical Fitness Differences between Freestyle and Greco-Roman Junior Wrestlers

**DOI:** 10.2478/hukin-2014-0052

**Published:** 2014-07-08

**Authors:** Erkan Demirkan, Mehmet Kutlu, Mitat Koz, Mehmet Özal, Mike Favre

**Affiliations:** 1 Physical Education and Sports School, Hitit University, Çorum- TURKEY.; 2 Department of Physical Education and Sport, Education Faculty, the University of Kırıkkale, Kırıkkale- TURKEY.; 3 Sports Sciences Faculty, Ankara University, Ankara – TURKEY.; 4 Department of Sport - General Diretorate of Sport, Ankara – TURKEY.; 5 Director of Olympic Sports S&C, University of Michigan. USA

**Keywords:** Anaerobic power, aerobic capacity, body composition, speed, strength, wrestlers

## Abstract

The aim of the present study was to examine physical fitness differences between Freestyle and Greco-Roman junior wrestlers. One hundred twenty-six junior wrestlers, comprising 70 Freestyle and 56 Greco-Roman wrestlers, participated in this study. The somatic and physical fitness profile included body mass, body height, body mass index, body composition, flexibility, maximal anaerobic power of the legs and arms, aerobic endurance, hand grip strength, leg and back strength, and speed. No significant differences were found in the anthropometric and physical features between Freestyle and Greco-Roman wrestlers. The Greco-Roman wrestlers had a significantly higher level of relative leg power, peak arm power, relative peak arm power, and relative average arm power than Freestyle wrestlers (p < 0.05). Greco-Roman wrestlers were significantly faster, had better agility, and had a greater level of leg strength than Freestyle wrestlers, but Freestyle wrestlers were more flexible than Greco-Roman wrestlers (p < 0.05). Discriminant function analysis indicated that peak arm power, agility, speed, and flexibility were selective factors for the differences between Freestyle and Greco–Roman wrestlers. In conclusion, the present study indicates that the differences between these wrestling styles promote physical fitness differences in elite wrestlers. The results reflect specific features of each wrestling style.

## Introduction

Wrestling is a sport discipline that places great demands on athletes in terms of physical preparation (Sterkowicz-Przybycień et al., 2011). Two wrestling styles, Freestyle and Greco-Roman, are included for men in the Olympics. Freestyle wrestling includes upper and lower body wrestling and is characteristic of short duration, high-intensity intermittent effort that lasts a total of 6 min for senior and junior wrestlers (2 × 3-min bouts). Anaerobic power is crucial because of the scoring system for Freestyle wrestling, which uses explosive techniques that may end the match before regulation time (Cipriano, 1993; Mirzaei et al., 2009). Greco-Roman wrestling allows only upper body moves and also has a bout duration of 6 min (2 × 3-min bouts) for senior and junior wrestlers according to new official rules. The sports level clearly differentiates the results of tests of strength endurance of arm and trunk muscles, of which function is extremely important in wrestling (Sterkowicz and Starosta, 2005). The importance of lower and upper body power lies in the ability to lift the opponent during offensive maneuvers and resist attacks while in defense. Elite wrestlers have been characterized by high maximal power output of the arm and leg muscles (Yoon, 2002).

Only a few studies have compared the physical fitness characteristics of both wrestling styles (Horswill, 1992; Baić et al., 2007; Gullón et al., 2011). Therefore, the present study compared the level of physical fitness between Freestyle and Greco-Roman elite wrestlers. Our hypothesis was that the official rule differences between the Freestyle and Greco-Roman styles promote physical fitness differences based on technical implementation in wrestling, in which the implementation of the different styles of wrestling partially influences the physical fitness of wrestlers.

## Material and Methods

### Subjects

One hundred twenty-six junior competitive wrestlers, 56 Greco-Roman wrestlers (16.4 ± 0.7 years old) and 70 Freestyle wrestlers (16.5 ± 0.6 years old), volunteered to participate in the study. The sample was composed of the best junior wrestlers in Turkey. All of the participants were invited to the national team camp. Before participating in the study, the parents of the subjects read and signed an informed consent form. The athletes were asked not to participate in daily training programs within 24 h prior to testing. Testing was completed for all of the wrestlers in the same laboratory and field facilities on three consecutive days. The subjects and coaches were informed about the experimental procedures and possible risks and benefits of the project. The study complied with the Declaration of Helsinki and was approved by the Bioethics Commission of the University of Ankara in Turkey.

### Body height and body mass

Body height and body mass measurements were made using a digital scale (Seca 664, Hamburg, Germany) with the subjects barefoot and wearing only shorts.

### Body composition

Body composition was determined by measuring the skinfold thickness on three parts of the body (subscapular, triceps, abdominal) with a Holtain caliper. Skinfolds were measured three times at each site to the nearest 0.5 mm, and the mean value was recorded. All skinfold measurements were taken on the right side of the body. Body fat content was calculated from the formula developed by Lohman (6). Fat-free mass was calculated by subtracting the fat tissue mass (in kg) from the total body mass.

### Anaerobic power and capacity

Wingate (WAnT) tests were used for the arms and legs during separate sessions. The lower-limb Wingate test consisted of 30-s supramaximal cycling against a resistance load of 75 g·kg^−1^ of body mass. Each test was performed on a Monark cycle ergometer (Model 894-E). Verbal encouragement was given to motivate the participants during the test (Inbar et al., 1996).

Arm cranking was performed with a standing body posture using a Monark 894E ergometer. Resistance load of 55 g·kg^−1^ of body mass was used for the upper limbs. The total number of revolutions performed during the entire 30 s test was counted, and power was calculated using a computerized MCE system, version 4.5 (JBM, Poland; Inbar et al., 1996). Verbal encouragement was given to motivate the participants during the test. During both arm cranking and leg cycling, mean power was defined as the average power generated during the 30 s interval (Hübner-Woźniak et al., 2004).

### Sprint test

After a standardized 15-min warm-up (low-intensity running, several acceleration runs, and stretching exercises), the subjects underwent a sprint test that consisted of two maximal 30 m sprints with timing at 10 and 30 m, with a 3 min rest period between each sprint.

### Maximal hand grip and leg-back strength

Each subject’s grip strength was measured for each hand using a Dynamometer (Takei A5001 Hand Grip Dynamometer, Tokyo, Japan). The average of two trials was recorded. Maximal leg and back strength (BS) was measured using a back muscle dynamometer (Takei A5002 Back and Leg Dynamometer, Tokyo, Japan). The average of two trials was recorded.

### Flexibility

Flexibility of the trunk was determined using a standard sit and reach test (Eveque, Sit and Reach bench, Cheshire, England). The recorded score for this test was the average of two trials.

### Aerobic endurance

Aerobic endurance was determined in a 20 m shuttle run test. The wrestlers started running back and forth on a 20 m course and touched the line at the end. The initial speed was 8.0 km/h, which was increased by 0.5 km/h every minute, in accordance with a pace dictated by a sound signal on an audiotape. The wrestlers were instructed to keep pace with the signal for as long as possible. When the subjects could no longer follow the pace, the last stage recorded was used to predict VO2max. A predicted VO2max was obtained using the equation of Leger and Gadoury (1989).

### Data analysis

The general characteristics of the participants are presented as means and standard deviations (SD). The differences between the Freestyle and Greco-Roman wrestlers were determined using an independent *t*-test. Additionally, discriminant function analysis was performed to determine which set of variables most accurately predicted wrestling styles. The level of significance for all of the statistical analyses was *p* < 0.05.

## Results

The physical characteristics and training experience of the Freestyle and Greco-Roman wrestlers are presented in Table 1. No significant differences were found between the characteristic features of Freestyle and Greco-Roman wrestlers.

The arm and leg anaerobic power and capacity of the Freestyle and Greco-Roman wrestlers are presented in Table 2. The Greco-Roman wrestlers had significantly higher relative average leg power (W/kg), peak arm power (W), relative peak arm power (W/kg), and relative average arm power (W/kg) than Freestyle wrestlers (*p* < 0.05; Table 2).

Aerobic endurance, speed, agility, strength, and flexibility in Freestyle and Greco-Roman wrestlers are presented in Table 3. The Greco-Roman wrestlers were significantly faster (10 m speed test) and more agile, had higher leg strength, and were more flexible than Freestyle wrestlers (*p* < 0.05; Table 3).

Discriminant analysis was used to test the differences between the Freestyle and Greco-Roman elite adolescent wrestlers with regard to physical fitness variables. Five variables were put into the classification function of the discriminant function procedure: peak arm power, agility, speed (both 10 and 30 m), and flexibility. These variables resulted in a final Wilks’ lambda of 0.386 (*p* < 0.05) and an average squared canonical correlation of 0.78, indicating that these variables accounted for approximately 61% (0.784) of the variance in predicting group membership. This model correctly classified 49 of 56 subjects for Greco-Roman wrestlers and 61 of 70 subjects for Freestyle wrestlers, with an overall prediction accuracy of 87.5% (Table 4).

## Discussion

The primary findings of this investigation indicated that elite level Greco-Roman and Freestyle junior wrestlers presented similar training backgrounds, body composition, and anthropometrical characteristics (Table 1). These results are consistent with previous studies that reported no differences in any of the anthropometrical and physical characteristics between the Freestyle and Greco-Roman groups (Horswill, 1992; Demirkan et al., 2011; Gullón et al., 2011). However, the present results confirmed a statistically significant difference in anaerobic arm power and capacity between the Greco-Roman and Freestyle wrestlers, but anaerobic leg power (with the exception of average relative leg power) and capacity were similar (Table 2). These results also showed that top-level Greco-Roman wrestlers had a higher level of anaerobic power and capacity (14.3% peak arm power [W], 14.7% relative peak arm power, and 9.8% relative average peak arm power) in the upper extremities than Freestyle wrestlers. These changes were most likely related to the fact that the competitors performed dynamic moves during both training and wrestling combat (i.e., lifting, throwing, and resisting opponents, which require upper body power), and all of the techniques in Greco-Roman wrestling must be performed with the upper body. Consequently, according to our results, we may conclude that good physical preparation of the upper extremities in Greco-Roman wrestlers is not only important but also a result of the long-term drilling of technical-tactical elements during the training process.

Scarce data are available on the physiological differences among elite Greco-Roman and Freestyle wrestlers. Horswill et al. (1992) found no significant differences in the mean and peak lower and upper limb power attained during a 30 s Wingate test between wrestlers of both styles. Gullon et al. (2011) found that no significant differences in the crank-arm Wingate test, either absolute or normalized to fat-free mass, between Freestyle and Greco-Roman wrestlers. This may be attributable to the grouping of variables (i.e., age, elite-nonelite, and sport experience). However, this investigation found no significant differences in aerobic endurance between Freestyle and Greco-Roman wrestlers. These results appear to be consistent with previous studies (Horswill, 1992; Gullón et al., 2011). Aerobic performance may be a basic requirement for wrestlers because a high level of aerobic power allows the athlete to maintain a high intensity of activities during a match and provides effective recovery during the 30 s rest period between the two 3-min rounds.

Significant differences in speed, agility, and flexibility were detected between the wrestling styles (Table 3). The statistical analysis indicated that Greco-Roman wrestlers were faster (6.3%) and more agile (5.5%) than Freestyle wrestlers. However, Freestyle wrestlers were more flexible (11.8%) than Greco-Roman wrestlers. Additionally, the discriminant function analysis revealed that the significantly different variables were peak arm power (W/kg), agility, speed (10 and 30 m), and flexibility among both wrestling styles (Table 4). According to these results, Greco-Roman wrestlers had higher peak arm power (693 ± 218 W), were faster in 10 m (1.74 ± 0.1 s), and were more agile (14.6 ± 0.6 s) than Freestyle wrestlers (594 ± 173 W, 1.85 ± 0.1 s, and 15.4 ± 0.8 s, respectively), but Freestyle wrestlers were more flexible (34 ± 7.0 cm) and faster in 30 m (4.30 ± 0.3 s) than Greco-Roman wrestlers (30 ± 6.2 cm and 4.39 ± 0.2 s, respectively). In contrast to these results, Baic et al. (2007) indicated that top-level Freestyle wrestlers had a higher level of strength endurance of the trunk and upper extremities than Greco-Roman wrestlers, based on discriminant function analysis. These authors assumed that their results were influenced by the specific features of each wrestling style. In another study, Gullon et al. (2011) compared both wrestling styles and found no differences in 10 m sprint times.

Numerous studies have been conducted to investigate differences between successful and less successful wrestlers and between male and female wrestlers (Roemmich and Frappier, 1993; Hübner-Woźniak et al., 2004; Vardar et al., 2007; Abellán et al., 2010; Pallares et al., 2011; Pallares et al., 2012). In one of these studies, Roemmich and Frappier (1993) employed discriminate function analysis to determine which collection of variables most accurately predicted wrestling success. They found that grip strength of the left hand, flexibility of the lower back and hamstrings, push-ups, strength of the right quadriceps, and total distance covered during a 12-min run were important in predicting wrestling success. In another study, Palleres et al. (2011) used binary logistic regression analysis to predict the probability of being an elite wrestler. They found that training experience, fat-free mass, one repetition maximum (1RM) strength and muscle power in the bench press and full squat, and peak power were selective factors. Palleres et al. (2012) used regression analysis and found that fat-free mass and 1RM strength were the most significant factors of successful female wrestling performance.

Superior upper body strength and anaerobic capacity in Greco-Roman wrestling might be more beneficial for the initiation of attacks and explosive execution of wrestling techniques because only upper body moves are allowed. This may stimulate the development of anaerobic power and capacity of the upper body. However, Freestyle and Greco-Roman wrestlers had similar lower body anaerobic power and capacity. Freestyle is a complex wrestling style that allows actions of both the upper and lower parts of the body. It requires the strength and power of both body parts. These results indicate that the increasing of upper body power requires also the developed lower body power.

## Conclusion

Freestyle and Greco-Roman wrestlers have similar characteristic features (age, body height, body mass, fat percentage, fat-free mass, and body mass index) and sports experience, but Greco-Roman wrestlers have a higher level of anaerobic upper body power and capacity than Freestyle wrestlers. Greco-Roman wrestlers perform dynamic moves (e.g., lifting, throwing, and resisting opponents) that require upper body power, and all of the techniques in Greco-Roman wrestling must be performed with the upper body, both in competitions and training.

## Figures and Tables

**Table 1:** Characteristic features of Freestyle and Greco-Roman wrestlers

	**Freestyle**	**Greco-Roman**
Age	16.5±0.6	16.4±0.7
Body height (cm)	170±8.0	170±8.0
Body mass (kg)	68.0±14.0	67.3±16.9
Fat %	9.0±4.9	8.7±6.4
FFM (kg)	61.3±9.6	60.5±11.2
BMI	23.2±3.1	23.0±4.0
Training experience (years)	5.7±1.6	5.3±1.5

p > 0.05. FFM, fat-free mass; BMI, body mass index.

**Table 2:** Comparison of anaerobic upper and lower limb power and capacity

**Variables**	**Freestyle**	**Greco-Roman**	***p***
Peak leg power (W)	895±210	906±250	0.77
Relative peak leg power (W/kg)	13.2±2.0	13.5±1.6	0.43
Average leg power (W)	461±100	478±119	0.39
Relative average leg power (W/kg)	6.8±0.8	7.1±0.6	0.02
Peak arm power (W)	594±173	693±218	0.01
Relative peak arm power (W/kg)	8.7±2.0	10.2±1.8	0.00
Average arm power (W)	316±94	348±96	0.06
Relative average arm power (W/kg)	4.6±0.9	5.1±0.6	0.00

p < 0.05. W: watt.

**Table 3:** Comparison of aerobic endurance, speed, agility, strength, and flexibility

**Variables**	**Freestyle**	**Greco-Roman**	***p***
VO2max (ml·kg·min^−1^)	50.1±6.3	51±4.9	0.42
10 m speed (s)	1.85±0.1	1.74±0.1	0.00
30 m speed (s)	4.30±0.3	4.39±0.2	0.05
Agility (s)	15.4±0.8	14.6±0.6	0.00
Right hand strength (kg)	43.9±9.1	45.7±9.3	0.28
Left hand strength (kg)	43.4±8.8	44.6±9.0	0.47
Back strength (kg)	148±39	154±26	0.37
Leg strength (kg)	180±40	204±32	0.00
Flexibility (cm)	34±7.0	30±6.2	0.01

p < 0.05.

**Table 4:** Standardized canonical discriminant function coefficients

**Variables**	**Function coefficients**	**Freestyle**	**Greco-Roman**
Peak arm power (W/kg)	−0.534	8.7±2.0	10.2±1.8
Agility (s)	0.684	15.4±0.8	14.6±0.6
10 m speed (s)	0.705	1.85±0.1	1.74±0.1
30 m speed (s)	−1.163	4.30±0.3	4.39±0.2
Flexibility (cm)	0.322	34±7.0	30±6.2
